# Blood Contamination in CSF and Its Impact on Quantitative Analysis of Alpha-Synuclein

**DOI:** 10.3390/cells9020370

**Published:** 2020-02-05

**Authors:** Katalin Barkovits, Niels Kruse, Andreas Linden, Lars Tönges, Kathy Pfeiffer, Brit Mollenhauer, Katrin Marcus

**Affiliations:** 1 Faculty of Medicine, Medizinisches Proteom-Center, Ruhr-University, 44801 Bochum, Germany; katalin.barkovits@rub.de (K.B.); andreas.linden@mpibpc.mpg.de (A.L.); kathy.pfeiffer@rub.de (K.P.); 2 Institute of Neuropathology, University Medical Center Goettingen, 37075Goettingen, Germany; n.kruse@med.uni-goettingen.de; 3 Department of Neurology, Ruhr-University Bochum at St Josef-Hospital, 44791 Bochum, Germany; lars.toenges@rub.de; 4 Paracelsus-Elena Klinik, 34128 Kassel, Germany; brit.mollenhauer@paracelsus-kliniken.de; 5 Department of Neurology, University Medical Center Goettingen, 37075 Goettingen, Germany

**Keywords:** cerebrospinal fluid, blood contamination, mass spectrometry, ELISA, urine strip, alpha-synuclein, quantification

## Abstract

Analysis of cerebrospinal fluid (CSF) is important for diagnosis of neurological diseases. Especially for neurodegenerative diseases, abnormal protein abundance in CSF is an important biomarker. However, the quality of CSF is a key factor for the analytic outcome. Any external contamination has tremendous impact on the analysis and the reliability of the results. In this study, we evaluated the effect of blood contamination in CSF with respect to protein biomarker identification. We compared three distinct measures: Combur10-Test^®^ strips, a specific hemoglobin ELISA, and bottom-up mass spectrometry (MS)-based proteomics for the determination of the general blood contamination level. In parallel, we studied the impact of blood contamination on the detectability of alpha-synuclein (aSyn), a highly abundant protein in blood/erythrocytes and a potential biomarker for Parkinson’s disease. Comparable results were achieved, with all three approaches enabling detection of blood levels in CSF down to 0.001%. We found higher aSyn levels with increasing blood contamination, highlighting the difficulty of authentic quantification of this protein in CSF. Based on our results, we identified other markers for blood contamination beyond hemoglobin and defined a grading system for blood levels in CSF samples, including a lower limit of tolerable blood contamination for MS-based biomarker studies.

## 1. Introduction

Cerebrospinal fluid (CSF) is one of the main sources for the discovery of biomarkers in the field of central nervous system diseases. It is produced in the choroidal plexus and surrounds the brain as well as the spinal cord; hence, CSF is in direct contact with the central nervous system. Changes in brain metabolism or homeostasis are most likely reflected in this body fluid. For diagnostic purposes, CSF is collected by routine lumbar punctures. During this procedure, unwanted vascular bleeding may occur, causing peripheral blood to contaminate CSF, which happens in up to 20% of standard lumbar punctures [1]. Those blood contaminations can introduce variabilities affecting reliable detection and/or measurement of biomarkers such as alpha-synuclein (aSyn) or routine CSF results like total protein measurements, as well as blood-CSF barrier function [2,3]. ASyn has been shown to be highly aggregated in the brains of Parkinson’s disease (PD) patients, and the associated reduction of aSyn in the CSF of PD patients might be used for disease diagnosis (reviewed in Reference [4]). The tremendous overlap of single values hampers the diagnostic utility of this marker, and factors that contribute to this variability therefore need attention. Besides aSyn detection in the brain, high amounts can be found in the bone marrow, kidney, bladder, and blood, especially in erythrocytes (https://www.proteinatlas.org/ENSG00000145335-SNCA/tissue) [5]. Consequently, the presence of only minimal blood portions in generated CSF samples will lead to an increased overall aSyn level, and the actual level of aSyn released from brain due to neurodegenerative processes cannot be assessed. Moreover, blood contamination has a substantial effect on the CSF protein composition as well as detection of low-abundance CSF- and/or brain-specific proteins [3,6]. These can be masked by highly abundant blood proteins, which makes it difficult to identify disease-relevant molecules with presumably low quantities. Aasebo et al. examined the impact of blood contamination on the global CSF proteome and showed that among 814 quantified proteins, the abundance of 262 was strongly affected [7]. In particular, proteins known to be blood-originating were the most affected, like purine nucleoside phosphorylase or platelet factor 4. In order to reduce the pre-analytical factor of blood contamination, several recommendations have been published by applying cut-off values either for the level of hemoglobin or for the red blood cell count (RBC). In brief, hemoglobin cut-off values ranging from <200 ng/mL to <1000ng/mL have been used as a measure of blood contamination [8,9]. Until 2012, a value of <500 RBC/µL was recommended, and this was later reduced to a 10-fold lower value of <50 RBC/µL [10,11]. However, determination of RBCs is only correct if cells are not lysed in the CSF during the time between sample collection and cell counting. Hence, this knowledge is highly important in order to reject blood-contaminated CSF samples before complex and expensive analyses like mass spectrometry are performed. One possibility to verify blood contamination is a combination of hemoglobin concentration determination by, for example, ELISA (enzyme-linked immunosorbent assay) and RBC. Nevertheless, approaches like ELISA are not available in all diagnostic laboratories and are expensive. An alternative for CSF could be the use of routine indicators from urine-strip-based analysis, which includes determination of various analytes, including hemoglobin detection. One test system, the Combur10-Test^®^ from Roche, was recently suggested to be applied for clinical analysis of CSF [12,13,14]. In particular, the strip was used to analyze levels of proteins, glucose, and leukocytes in CSF with sensitivities and specificities of over 90% compared to standard laboratory analysis, indicating this test strip to be a simple, robust, and accurate diagnostic tool. For RBC detection, a urine-strip-based approach using the Multistix system from Bayer was shown to be highly sensitive for detection of blood in CSF, albeit with a high false positive rate [15,16].

With our study, we aimed to find a simple, cost-effective, and robust method for the evaluation of blood content in CSF as an upstream tool prior to mass spectrometric CSF proteome analysis. Using artificially blood-contaminated CSF, we investigated the Combur10-Test^®^ systems’ applicability to determination of different blood levels and compared the results with results from hemoglobin ELISA- and mass spectrometry (MS)-based analysis. We found that the Combur10-Test^®^ and MS analysis were useful approaches to detect blood contamination down to a level of 0.001%, which is below the recommended cut-off value of <50 RBC/µL. For global CSF proteome analysis, as well as reliable quantification of CSF aSyn, we found levels down to 0.01% to be tolerable.

## 2. Materials and Methods

### 2.1. Sample Preparation

Single-use aliquots of CSF samples, collected by routine lumbar puncture from patients with normal pressure hydrocephalus were randomly selected from the Kassel cohort I (institutional review board (IRB) number: FF89/2008) and from patients with a neurodegenerative disease (Bochum cohort; IRB number: # 17-6119), were all processed as previously described [17]. Whole blood from a healthy volunteer was collected by venous puncture in ethylenediaminetetraacetic acid (EDTA) tubes, stored at −80 °C in 0.5 mL aliquots and used for spiking experiments.

Spiking experiments: 1% whole blood was spiked into CSF aliquots and further diluted with CSF from the same aliquot to obtain final blood concentrations of 0.1%, 0.05%, 0.001%, and 0.0001%. aSyn concentration in the spiked samples were determined at 1 in 8 dilutions, while for quantification of hemoglobin, the CSF samples were diluted 1 in 100,000. Endogenous aSyn and hemoglobin concentrations were determined in non-spiked aliquots diluted 1 in 8 and 1 in 100, respectively. The study was conducted according to the Declaration of Helsinki, with informed written consent provided by all subjects.

### 2.2. Immuno-Based Quantification of Hemoglobin and aSyn

For the quantification of hemoglobin, antibody A80-134A (Bethyl Labratories, Montgomery, TX, USA) diluted to 1 µg/mL was used for both capturing and detection. The best concentration for A80-134A as capture antibody was determined by 1 µg/mL, 2 µg/mL, and 4 µg/mL of A80-134A together with 1 µg/mL SulfoTAG-labeled A80-134A as detection antibody. The lower limit of detection and the Hillslope with 1 µg/mL A80-134A gave the best results. Next, we titrated SulfoTAG-labeled A80-134A as detection antibody at concentrations of 0.5 µg/mL, 1 µg/mL, and 2 µg/mL together with 1 µg/mL A80-134A as capture antibody, showing best results with 1 µg/mL. CSF samples were diluted 1 in 100. Blood samples artificially spiked with blood were measured at a 1 in 100,000 dilution to meet the quantification range of the calibration curve.

Quantification of aSyn was done as previously described [18].

### 2.3. Combur10-Test^®^ Strip Analysis

The Combur10-Test^®^ analysis serves as a semi-quantitative measurement tool for hemoglobin or erythrocytes, and utilizes a colorimetric reaction which is caused by the oxidation of tetramethylbenzidine after catalysis of hydroperoxide by hemoglobin. It was performed according to the manufacturer’s instructions (http://www.cobas.com/content/dam/cobas_com/pdf/product/Combur-Test-strip/combur%20test%20strip%20brochure.pdf). Briefly, the test strip was incubated with 50 µL CSF for 1 s and the visual inspection was performed 1 min later. The semi-quantitative evaluation was done by comparing the color reaction of the sample with the manufacturer’s classification of Hb/erythrocyte (Ery) content in the following ranges: negative; + (~ 10 Ery/uL), ++ (~ 25 Ery/µL), +++ (~ 50 Ery/µL), ++++ (~250 Ery/µL).

### 2.4. Protein Digestion

Digestion was performed as reported in Reference [6] with some modifications. Measures of 20 µL CSF were mixed with 50 µL 0.1% Rapigest™ (Waters, Eschborn, Germany) in 50 mM NH_4_HCO_3_ and dithiothreitol (DTT) (final concentration 5 mM) and heated at 60 °C for 30 min, followed by cooling down at room temperature for 10 min. Alkylation was performed with 15 mM iodoacetamide (IAA) (final concentration) and incubated in the dark for 30 min at room temperature. Tryptic digestion (trypsin in 10 mM HCl and 50 mM NH_4_HCO_3_) overnight at 37 °C in a thermomixer was performed with a trypsin:protein ratio of 1:50. Digestion was stopped by adding 10% TFA solution (to a pH < 2) and samples were incubated for 45 min at 37 °C. Centrifugation was performed at 10,000 rpm for 10 min at room temperature and the supernatant was transferred into a new Eppendorf tube. The peptide concentration was determined by amino acid analysis as previously described [19].

### 2.5. Nano LC-MS/MS Analysis

For MS-based analysis, a nanoHPLC system Ultimate 3000 ((Thermo Fischer Scientific, Bremen, Germany)) on-line coupled to a quadrupole-orbitrap mass spectrometer (Q Exactive; Thermo Fischer Scientific, Bremen, Germany) was used. First, 500 ng tryptically digested sample was loaded for 10 min on a C18 trap column (PepMap100 C18, 100 µm ID × 2 cm, particle size 5 µm and pore size 100 Å; Thermo Scientific) with 0.1% TFA and 30 µL/min flow rate. Afterwards, the trap column was connected online to an analytical C18 column (PepMap C18, 75µm × 50 cm, particle size 2 µm, pore size 100 Å; Thermo Scientific). Separation was performed with a flow rate of 400 nL/min using the following solvent system: (A) 0.1% formic acid (FA); (B) 84% acetonitril (AcN), 0.1% FA. Peptide separation was performed from 5–40% B within 180 min. Full MS spectra were scanned between 350–1400 *m*/*z* with a resolution of 70,000 at 200 *m*/*z* (AGC target 3e6, 80 ms maximum injection time). Capillary temperature was set to 250 °C and spray voltage to 1600 V (positive mode). Lock mass polydimethylcyclosiloxane (*m*/*z* 445.120) was used for internal recalibration. The *m*/*z* values initiating MS/MS were set on a dynamic exclusion list for 30 s and the 10 most intensive ions (charge 2+ to 5+) were selected for fragmentation. MS/MS fragments were generated by higher-energy-collision-induced dissociation and the fragmentation was performed with 27% normalized collision energy. The fragment analysis was performed in an orbitrap analyzer with resolution 35,000 at 200 *m*/*z* (AGC 1e6, maximum injection time 120 ms). After each sample measurement, a 50 min washing step gradient was included using 5% to 95% of B.

### 2.6. Protein Identification and Quantification

Protein identification was performed using Proteome Discoverer software (ver. 1.4.1.14) (Thermo Fisher Scientific). Thermo raw files were imported and matched against UniProt/Swiss-Prot using human taxonomy (released 2016_11, 552,884 sequences entries in the whole database and 20,121 for human) using the Mascot search algorithm (ver. 2.5.1) (Matrix Science Ltd., London, UK). Database search was performed with the following parameters: mass tolerance 5 ppm for precursor and 20 mmu for fragment ions, missed cleavages 1, modifications methionine oxidation as dynamic and cysteine carbamidomethylation as fixed, FDR threshold <1%.

Label-free quantification was performed by using Progenesis QI software (Nonlinear Dynamics Ltd., Newcastle upon Tyne, UK). Raw files generated by the mass spectrometer were imported into the software and all runs were matched to the most suitable run among them (by automatic selection). Afterwards, the software generated a list of features including the *m*/*z* values of all measured peptides at a given retention time. The following filters were used at feature level: allowed charge state in the range 2+ and 5+, rejecting the features with two or fewer isotopes. The raw abundances of each feature were automatically normalized in order to correct experimental variations. Experimental setup was set to within subject comparison two groups. Quantified features were then matched to peptide and protein identification by importing the search results generated by proteome discoverer (see protein identification). Only unique peptides were used for quantification. The mass spectrometry proteomics data were deposited to the ProteomeXchange (version 2.4.11) Consortium via the PRIDE [20] partner repository with the dataset identifier PXD014515 and 10.6019/PXD014515

## 3. Results

**Figure 1:**
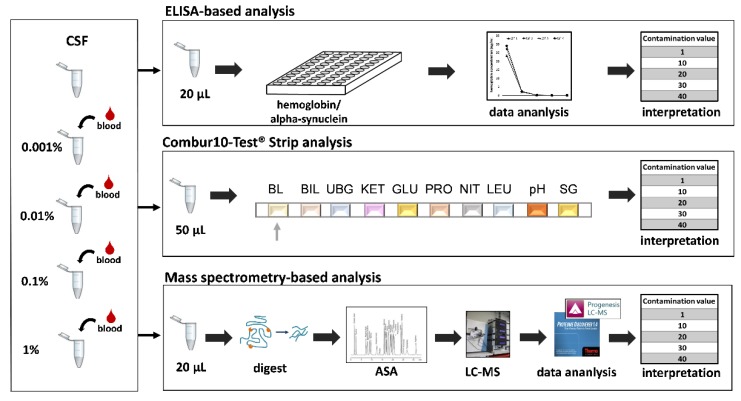
Overview of our study. Cerebrospinal fluid (CSF) from four different individuals was artificially spiked with four different concentrations of blood ranging from 0.001% to 1%. Those samples were subsequently analyzed using three independent methods including enzyme-linked immunosorbent assay (ELISA), Combur test strips, and MS-based bottom-up proteomics.

In this study, we evaluated the performance of urine reagent test strips for the detection of blood-contaminated CSF prior to mass spectrometry (MS)-based analysis as a fast and cost-effective approach alongside the currently performed RBC (red blood cell) count in clinical laboratories. The Combur10-Test^®^ (Roche) strip-based analysis includes determination of the RBC numbers as well as the hemoglobin content, which allows for detection of intact and lysed blood cells in parallel. The results were compared to RBC counts as well as hemoglobin levels obtained by ELISA (enzyme-linked immunosorbent assay) in order to define a comprehensive categorization system for blood content in CSF. In particular, the lower limit of tolerable blood contamination for MS-based analysis regarding the global CSF composition and the detectability of CSF aSyn was investigated. The general study design is presented in Figure 1.

### 3.1. Detection of Blood Levels in CSF

To examine applicability of the Combur10-Test^®^ strip to detect CSF blood contamination, we first generated samples with different levels of blood. Therefore, we used CSF samples from four different individuals (CSF 1–4). The number of RBCs of these samples was in the range of 0 to 8 per µL, indicating that they were not blood contaminated. Blood in four different concentrations ranging from 0.001% to 1% was subsequently spiked into these samples in order to generate artificial blood-contaminated CSF samples with defined blood levels. After preparation, all samples were stored at −80 °C to simulate laboratory conditions. All samples were analyzed with a highly sensitive electrochemiluminescence-based hemoglobin ELISA established in our laboratory as a benchmark approach, as well as by Combur10-Test^®^ strips.

#### 3.1.1. Hemoglobin ELISA

**Figure 2:**
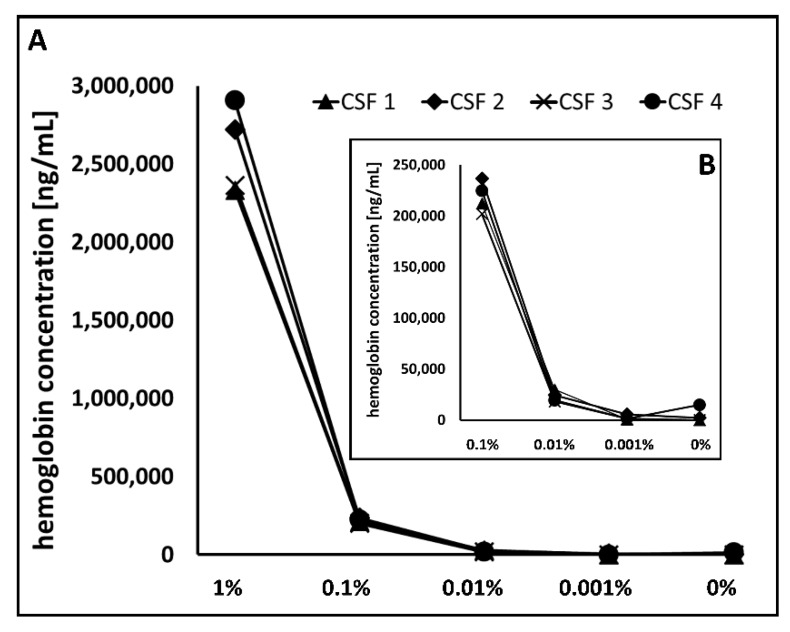
Blood-level-dependent hemoglobin concentration. (**A**) Hemoglobin concentration was determined in four CSF reference samples (0% blood) and corresponding artificially blood spiked samples in the range of 0.001% and 1% by ELISA. All measurements were performed in duplicate. (**B**) The magnified graph clearly demonstrates a strong increase in hemoglobin quantity starting from 0.01% spiked blood.

Hemoglobin is an established marker for the detection of blood in CSF since it is highly expressed in red blood cells [21]. The level of hemoglobin thus directly correlates with the amount of blood in a given sample. If a low RBC count but elevated hemoglobin level is observed in CSF samples, it can be assumed that RBC lysis occurred. Hence, detection of blood contamination solely by RBC count could leave introduced blood undetected due to cell lysis. The hemoglobin levels of the two reference samples, CSF 1 and 3, were below the lower detection limit, indicating that these two samples did not contain blood, which is consistent with the RBC values of 3 and 0 per µL, respectively (Figure 2). For CSF 2, with a RBC count of 0/µL, a hemoglobin concentration of 2143 ng/mL, and for CSF 4, with a RBC count of 8/µL, a concentration of 14,820 ng/mL was detected. From the artificially spiked samples with a blood level of 0.001%, the hemoglobin concentration ranged from 800 to 5555 ng/mL. Here, the hemoglobin level of CSF 4 was only 1435 ng/mL. A significant increase in hemoglobin concentration could be observed from a spike-in concentration of 0.01% (Figure 2 inset). For the samples with blood level of 0.01%, 0.1%, and 1% hemoglobin concentrations in the range of 18,130 to 20,820 ng/mL, 201,940 to 236,650 ng/mL, and 2,332,350 to 2,906,110 ng/mL were observed. In comparison to recommended cut-off values for hemoglobin concentrations of <1000 ng/mL as a measure of blood contamination [9], our reference samples (non-blood-contaminated CSF samples according to the RBC count) showed hemoglobin concentrations between 0 and 14,820 ng/mL.

#### 3.1.2. Combur10-Test^®^ Strip

The Combur10-Test^®^ strips were originally designed to test urine for blood content and have previously been shown to allow for the exclusion of blood-contaminated CSF samples [15,16]). This test enables both a semi-quantitative measurement of RBC numbers and the hemoglobin content in parallel. In our study, we used this test strip to detect blood in CSF. A visual evaluation on the basis of a given comparison scale was carried out according to the manufacturer’s instructions, enabling the detection of five different blood levels, including “negative”, corresponding to undetectable blood levels, and four stages, with 1+ (+), 2+ (++), 3+ (+++), and 4+ (++++) corresponding to increasing blood content. About 10 RBCs are expected for 1+, 25 RBCs for 2+, 50 RBCs for 3+, and over 250 RBCs for 4+. Overall, an increase of blood content corresponds to increasing amounts of spiked blood (Table 1). Among the four reference samples, three samples (CSF 1, 3, and 4) did not contain blood, as expected since all samples had RBC counts below 10 (based on the laboratory values). In contrast, CSF 2 was classified as level 2+, indicating the presence of blood, which was also reflected in the measurements of CSF 2 after blood spike-ins (Table 2). From a blood level of 0.1%, all samples were classified to grade 4+. Based on the recommended cut-off values for RBCs of <50 RBC/µL as a measure of blood contamination [11], samples with a grade of 3+ (corresponding to 50 RBC according to the strip) are defined as blood-contaminated samples corresponding to a blood level of 0.01%.

### 3.2. Definition of Blood Protein Markers by Mass Spectrometry

**Figure 3:**
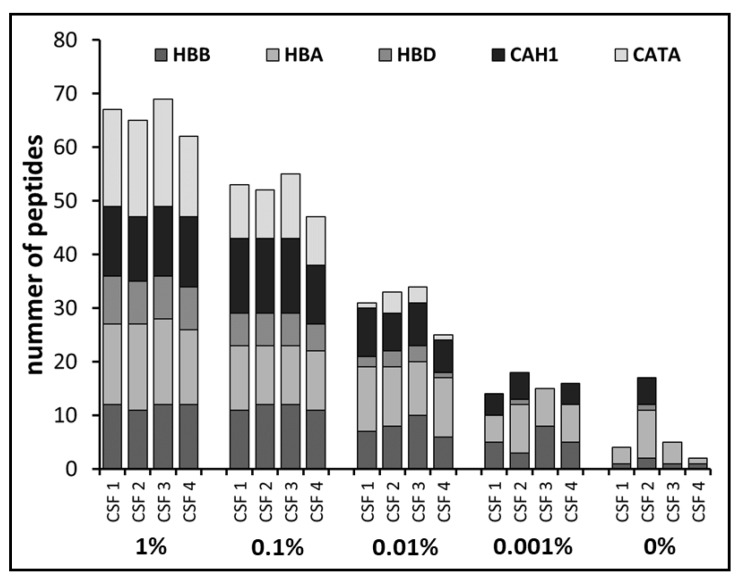
Identification of the selected blood proteins. Shown is the number of identified peptides for hemoglobin including subunit alpha (HBA), beta (HBB), and delta (HBD), carbonic anhydrase 1 (CAH1), and catalase (CATA) within the CSF samples of four different individuals (CSF 1 to CSF 4) and the respective blood spike-in levels, ranging from 0.001% to 1%. In all samples, hemoglobin (HB) was detected, while CAH1 and CATA were consistently identified in samples with increased blood levels. Furthermore, increasing blood levels led to enhanced peptide identification per protein.

One aim of our study was to evaluate the Combur10-Test^®^ strips as a tool for the simple detection of blood in CSF samples in order to exclude contaminated samples from further analyses. In modern proteomics, mass spectrometry is the method of choice and most of our protein biomarker studies are based on mass spectrometric applications. Consequently, we were interested in complementing our findings with MS data. MS analysis enables the simultaneous detection and quantification of several hundred up to thousands of proteins in complex samples [22,23]. For CSF proteome analysis, we used an established workflow, which results in the identification of over 700 proteins in CSF samples [6]. We examined the identification pattern of the three blood proteins hemoglobin (HB) (subunit alpha and beta), carbonic anhydrase 1 (CAH1), and catalase (CATA), which have previously been used as blood contamination markers in CSF proteomics [21]. As expected, we observed detection of the three selected proteins to be dependent on the amount of spiked blood within the samples. Hemoglobin (subunit A, HBA, and subunit B, HBB) was detected in all samples at all spike-in levels (Figure 3). Identification of CAH1 and CATA corresponded to increasing amounts of blood content. For example, in one of the reference samples (CSF 2), CAH1 but not CATA was detected. In the samples with a blood level of 0.001%, CAH1 was identified in almost all samples except for CSF 3, while CATA was not detected in any of those. Starting from a blood level of 0.01%, all three proteins were consistently identified in all samples. In addition to qualitative measurements of the three proteins, we further examined the number of identified peptides per protein in order to investigate whether there was a correlation between peptide numbers and blood level. Indeed, we observed higher numbers of identified peptides with increasing blood levels (Figure 3). In summary, this observation suggests that simultaneous detection of HB, CAH1, and CATA could serve as a direct indicator for blood contamination.

### 3.3. Categorization of CSF Blood Levels

An important aspect in the detection of blood contaminations is the knowledge of the blood content in order to give a statement about the degree of contamination. As described above, different blood levels could be successfully detected with ELISA, the Combur10-Test^®^ strips, and MS-based analysis. In order to be able to use these results for further classification of clinical samples, the next step was to establish a general categorization system for blood levels in CSF. We defined five different contamination values: (I) “negative” corresponds to 1, (II) very low to 10, (III) low to 20, (IV) high to 30, and (V) very high to 40 (for summary see Table 2). Specific values from each method were assigned to the respective categories. In brief, for ELISA concentration ranges, for the Combur10-Test^®^ strips, the grading according to the manufacturer, and for MS, the identification pattern of HB, CAHA1, and CATA were used (for detailed information see Table 2).

**Figure 4:**
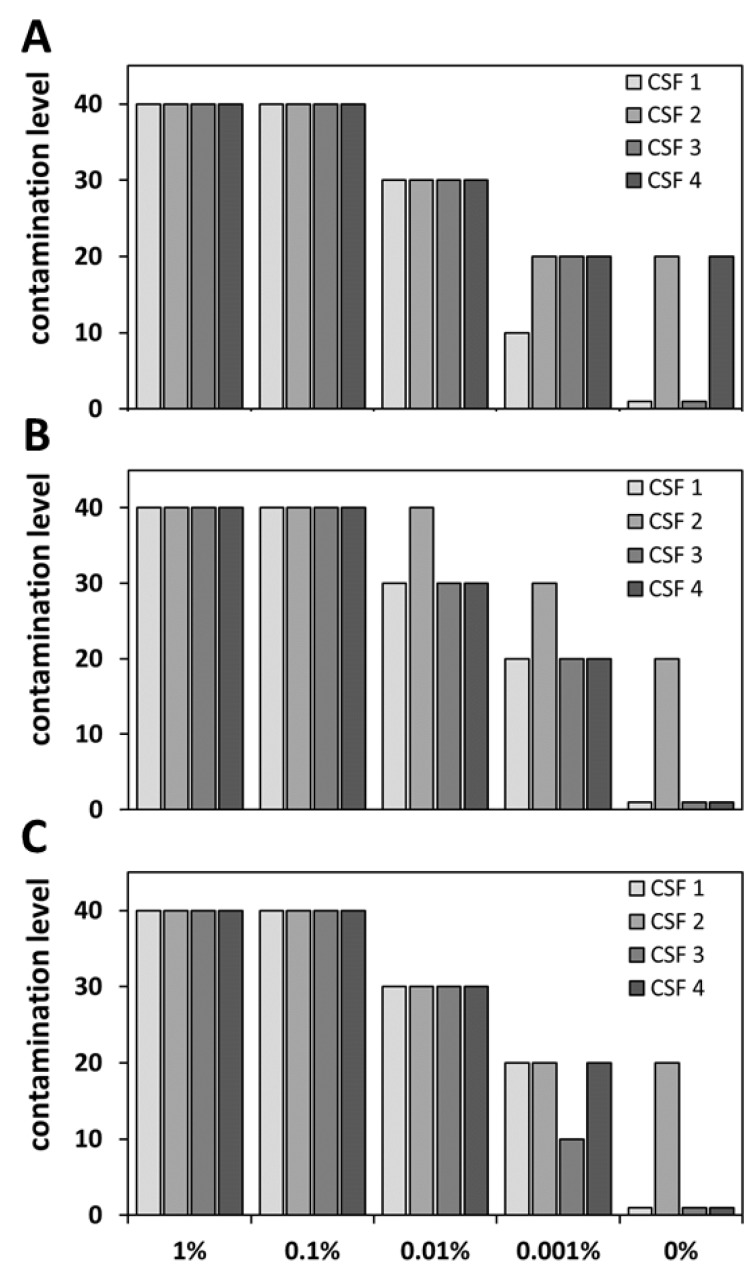
Application of the blood level categorization system to the results of the different methods. Shown are the classification results for ELISA (**A**), Combur10-Test^®^ Strips (**B**), and MS (**C**) depending on the defined contamination levels listed in Table 2. The results showed a high classification overlap for the different methods for samples with blood spike-in concentrations 1% to 0.01%.

This system was applied to the results of the three performed analyses from the four investigated CSF samples (CSF1 to CSF 4), including the artificial blood-contaminated CSF samples. Overall, the categorization of the different measurements showed great overlap for CSF with a blood spike-in content of 1%, 0.1%, and 0.01% (Figure 4). A contamination value of 40 was assigned to all samples from all measurements with 1% and 0.1% blood spike-in. A lower contamination value of 30 was obtained for all CSF samples with a blood level of 0.01%, expect for sample CSF 2 within the Combur10-Test^®^ analysis which exhibited a contamination value of 40. Decreased correlation rates between the different methods were found for the lowest spike-in concentration of 0.001% and for the reference samples (not artificially spiked with blood). Especially in the latter case, the sample CSF 4 was classified with a contamination value of 20 based on the ELISA results, whereas the sample was assigned a contamination value of 1 using the strip and LC-MS approaches. However, overall, comparable classification for the results of the three methods could be shown for samples with contamination values of 40 and 30. Based on the recommended cut-off values for RBCs, the contamination value 30 could be defined as blood contamination. Thus, according to our results, all methods can be used to identify blood contamination according to published guidelines [10,11].

In a next step, we applied the categorization system to 15 clinical CSF samples. Here, we aimed to evaluate the consistency of categorization for the different methods. Overall, the three approaches led to the same classification in nine samples (Table 3, for actual values see Supplementary Tables S1, S2, and S3). In summary, the Combur10-Test and LC-MS can be used for the categorization of CSF samples, since our results revealed a high consistency for these two independent approaches. Furthermore, they can be used for a clear identification of blood contamination and thus to identify samples which can be used for subsequent analyses. In terms of rapid, simple, and inexpensive determination of blood contamination, we recommend the Combur10-Test^®^.

### 3.4. Blood Contamination Influenced the Global CSF Proteome

**Figure 5:**
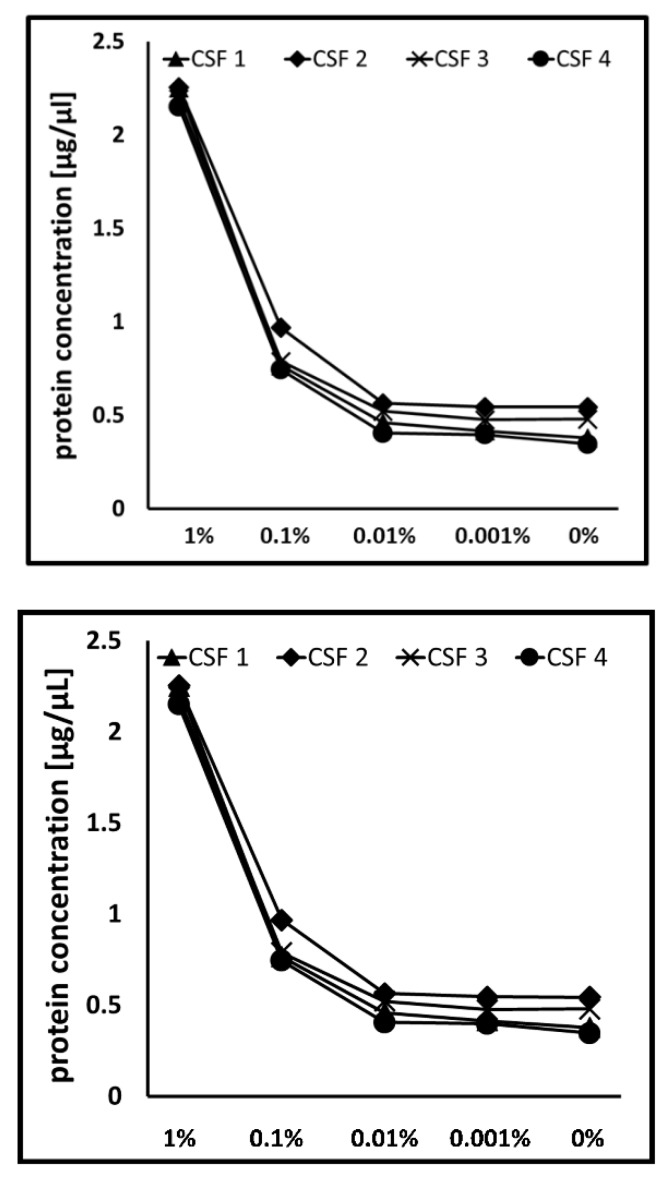
Comparison of the impact of different blood contamination levels on the protein concentration. Dependent on increased spike-in blood concentrations, increase of protein concentration was observed starting from the spike-in concentration of 0.1%.

In addition to the identification of CSF blood contamination, we further wanted to estimate the impact of blood spike-in concentrations on the CSF proteome. Changes in the proteome due to blood contamination can have an enormous impact on protein quantification. While equal protein concentrations were detected for samples with lower blood levels (0.01%, 0.001%, and 0%), an increase was observed from a spike-in concentration of 0.1% (Figure 5). Moreover, on protein identification level, significantly lower numbers of protein were detected with a blood level of 1% (data not shown).

For proteomics analysis, it is known that the overall variance is influenced by the analytical variance plus the biological variance [24]. Moreover, protein detectability and thus quantifiability are strongly dependent on the overall composition of the sample. Additionally introduced proteins due to, for example, blood contamination will consequently influence the analysis and lead to falsified results.

**Figure 6:**
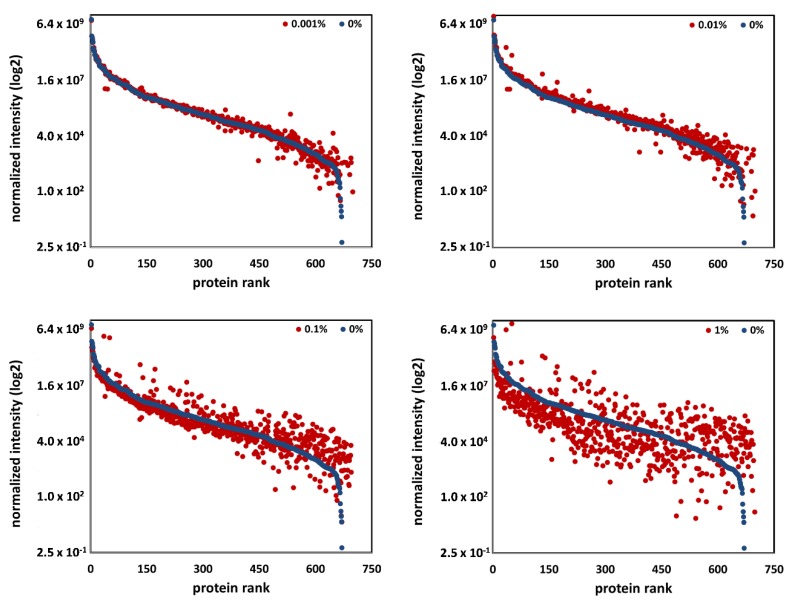
Quantitative impact of blood contamination on the global proteome. The normalized intensity values (obtained from the data analysis with the MS software Progenesis QI for CSF 1) of each protein in the reference sample (blue dots) were plotted in comparison to corresponding spike-in samples (red dots) including blood levels of 0.001%, 0.01%, 0.1%, and 1%. Decreases in the relative protein abundance were observed for samples starting with blood levels of 0.1% and 1%, while the number and abundance of non-specific CSF proteins increased.

In order to define a limit of tolerable blood contamination for a global quantitative proteome analysis, we investigated the impact of the different blood levels on protein quantification. Therefore, the data of all samples were processed with a label-free quantitative approach using Progenesis QI software. The normalized intensity values for all quantifiable proteins in each individual sample were calculated. We defined the proteome of samples with contamination value of 1 (CSF samples with 0% blood) as CSF reference proteome and all detected proteins as CSF-specific. Afterwards, we plotted the normalized intensity of each protein in the reference sample in comparison to corresponding spike-in samples (Figure 6, data shown exemplarily for CSF 1, data for CSF 2, CSF 3, and CSF 4 can be found in Supplementary Figure S1). Dependent on the respective blood level, an obvious decrease in the relative protein abundance was observed for samples starting with blood levels of 0.1% and 1%. Notably, the number and abundance of non-specific CSF proteins increased in parallel. Our results clearly showed that from a blood level of 0.1%, the protein pattern in the samples differed significantly from CSF reference samples (see Figure 6).

In summary, all our results demonstrated a significant effect of blood content on the detectability and quantifiability of CSF-specific proteins at already low levels. We suggest considering CSF samples to be blood-contaminated from a contamination value of 30 (blood levels of 0.1%). Such samples should be excluded from any further quantitative proteome analysis.

### 3.5. Impact of CSF Blood Contamination on aSyn Quantification

Blood contamination can introduce variations affecting the reliable detection and/or measurement of biomarkers such as aSyn [2]. Brain-released aSyn has been described as a potential biomarker for Parkinson’s disease [25]. Indeed, it is also present in high amounts in erythrocytes. Consequently, the presence of only minimal blood portions in generated CSF samples will lead to an increased overall aSyn level, and the actual level of aSyn released from brain due to neurodegenerative processes cannot then be assessed.

**Figure 7:**
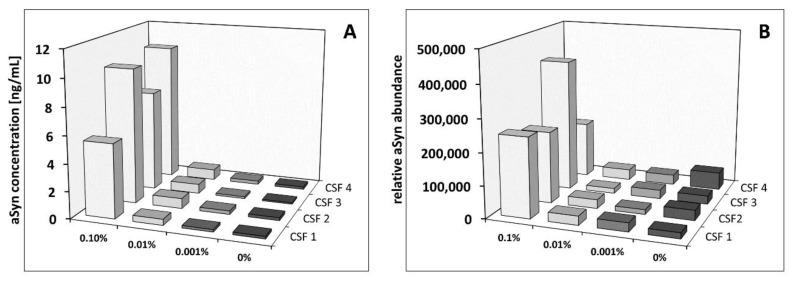
Influence of blood contamination on the quantification of aSyn using ELISA and label-free-based MS using artificially blood-contaminated CSF. Comparative quantification of aSyn in non- and artificially blood-contaminated CSF samples in the range of 0.001% to 0.1% from four different individuals (CSF 1 to CSF 4) was performed with ELISA (**A**) and MS-based analysis (**B**). Shown are in (A) the aSyn concentrations in ng/mL and in (B) the relative abundance obtained with the data analysis utilizing the MS software Progenesis QI.

We examined the impact of blood contamination on quantification of aSyn using our established ELISA as well as MS. With ELISA, aSyn was quantified in all samples. Comparable concentrations were observed for the CSF reference samples and the respective spike-in samples with a blood level of 0.001%. Starting from spike-in levels of 0.01%, elevated aSyn concentrations were detected (Figure 7). With blood levels of 0.1%, significant increases of aSyn were observed, showing a concentration range of 5 to 10 ng/mL compared to a concentration range of 0.1 to 0.2 ng/mL for the CSF reference samples. Thus, the aSyn concentration of these samples increased up to 100-fold. In contrast, an increase of aSyn abundance obtained with MS-based quantification was observed starting from a 0.1% blood content. Notably, reliable relative quantification of aSyn by MS, based on clear identification of the protein on peptide level, was only possible from a blood level of 0.1%. In summary, these data suggest that ELISA quantification of aSyn is biased by blood level from 0.01%, and for MS-based quantification starting from 0.1%.

**Figure 8:**
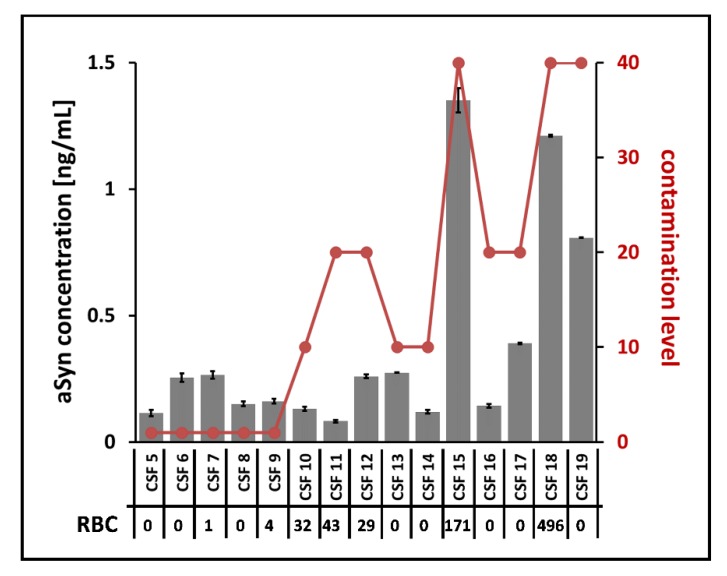
Correlation of ELISA aSyn quantification, contamination levels, and RBC counts from clinical samples. Shown are aSyn concentrations from 15 clinical samples (CSF 5 to CSF 19) obtained by ELISA analysis in relation to the detected contamination levels (marked in red) based on the Combur10-Test^®^ and to the RBC count from routine laboratory diagnostics. ELISA measurements were performed in duplicate. ASyn was quantified in all samples.

In addition to the artificially spiked CSF sample set, we inspected the aSyn concentration in the 15 clinical samples. Due to the low amounts of aSyn concentrations in these samples, a reliable quantification was exclusively possible using ELISA, since an unambiguous identification of the protein on peptide level was not possible expect for two samples when utilizing MS (data not shown). Using ELISA, aSyn could be quantified in all clinical samples within a concentration range of 0.08 to 1.4 ng/mL, while three samples showed noticeably elevated concentrations over 0.8 ng/mL. In order to correlate the obtained aSyn concentrations with possible introduction by blood contamination, we examined the individual contamination level determined with the Combur10-Test^®^ and the routine laboratory parameters of the RBC count. Among the samples, contamination levels in the range of 1 to 40 and RBC counts in the range of 0 to 496 per µL were observed (Figure 8). In particular, the three samples with comparatively high aSyn concentrations showed contamination levels of 40. While two of these samples had RBC counts over the recommended cut-off value of 50 RBC/µL (CSF 15 and CSF 18), no RBCs were detected for the third one (CSF 19), indicating that lysed RBCs were responsible for increased hemoglobin content. This might explain the observed elevated aSyn concentration within this sample, suggesting introduction of blood aSyn due to traumatic lumbar puncture. These results underline the use of the Combur10-Test^®^ strips as an additional application to identify blood contamination in addition to the routine determination of RBCs.

Overall, we demonstrated for the first time that the Combur10-Test^®^ can be used as an efficient tool to detect blood contamination on the one hand, and to determine tolerable blood levels in CSF samples on the other, enabling reliable results to be achieved in subsequent proteomic analyses.

## 4. Discussion

Proteins released from dying cells in the central nervous system due to degenerative processes might serve as biomarkers for various neurodegenerative diseases. Cerebrospinal fluid (CSF) surrounds the brain and functions to protect brain tissue from injury and ischemia, among other functions. Due to its proximity to the degenerating tissue, it is an optimal resource for protein biomarker identification in neurodegenerative diseases. CSF is derived from blood plasma, normally contains approximately 80% plasma proteins and a few white blood cells, and is free of red blood cells [26]. A general challenge of proteomics-based biomarker studies is the availability and use of appropriate samples. Reliable detection and quantification of a protein biomarker candidate might be strongly influenced by the presence of even low concentrations of sample contamination such as from chemical polymers or blood. This holds true for both the level of single proteins and the global protein CSF level. For example, aSyn, a key protein in the pathogenesis of PD [27] and potential diagnostic biomarker for this disease [28], is a highly abundant protein in blood cells. Blood contamination thus strongly influences the level of aSyn and alters its quantification [9]. We found a blood contamination level of >0.01% is critical for quantification of aSyn using LC-MS (Figure 8). On a global scale, Halbgebauer et al. provided an overview of global proteome studies for the detection of protein markers for PD [29]. The authors showed that most of the detected protein marker candidates were found only in a single study. Moreover, candidates detected more than once often showed variances in their regulation in independent studies. One reason discussed for the observed inconclusive results among the different studies was unconsidered CSF blood contamination. In summary, quantitative information of a protein should be assessed with caution if CSF samples are blood-contaminated. To find out what level of blood contamination is still acceptable for proteomic analysis, Aasebo et al. investigated the effect of different blood spike-in levels in CSF (0.5% and 2% blood) [7]. Quantified proteins were categorized in three groups: (i) unaffected proteins with less than 20% abundance change, (ii) uncertain abundance change of 20–50%, and (iii) affected abundance change of more than 50% compared to a non-spiked reference CSF sample. From their data, they concluded that levels of proteins from Category (i) are not affected by blood contamination. Quantification results of those proteins can be considered reliable and could be included for quantification even if samples are blood-contaminated. However, higher CSF blood levels (e.g., 2%) were supposed to lead to suppression of lower abundant proteins and hence their real abundances would not be reflected in label-free shotgun proteomics. RNA levels are also influenced by blood: Müller et al. revealed that the levels of several CSF microRNAs which were shown to have abnormal expression in Alzheimer’s disease (AD) patients suggested to have a role in AD pathogenesis were strongly affected by blood contamination [30,31]. Therefore, strict sample selection is essential. Our data showed that with a blood level of even 0.1%, a substantial suppression on the global proteome was obtained (Figure 7), and we recommend exclusion of CSF samples with blood levels > 0.1% from subsequent proteomic studies. We additionally checked different methods—Combur10-Test^®^, ELISA and mass spectrometry—and established a categorization system for fast and reliable blood determination in CSF. We found a contamination level of up to 30 (corresponding to a blood level of 0.01%) to be acceptable for subsequent proteomic analysis. Moreover, results of the Combur10-Test^®^ and mass spectrometry showed very good correlation (86%) (Table 2), underlining the applicability of the urine strips for a fast and simple evaluation of CSF sample quality for subsequent analyses such as global proteomics. We found that the RBC value did not reflect blood contamination if lysis of blood cells has occurred (e.g., CSF 19, Figure 8). In such cases, the Combur10-Test^®^ and mass spectrometry both reliably revealed the presence of blood in the sample. ELISA was indeed able to detect hemoglobin (HB) down to a level of 2143 ng/mL, but we observed high CVs of up to 25% for individual samples, preventing the estimation of true blood contamination levels. In comparison to previous studies [8,9] which defined HB <200 ng/mL to <1000ng/mL, we determined HB levels <100,000 ng/mL as an exclusion level for blood-contaminated CSF. Of note, the use of different ELISA systems could have had an influence on the results, as different antibodies can provide divergent results [27,32,33]. For example, no significant correlation between hemoglobin and aSyn values in CSF was observed in the studies by Goldman et al. and Kang et al., which currently does not allow strict HB cut-off values to be used [34,35]. Overall, it must be noted that the categorization system developed cannot provide an absolute quantitative statement about existing blood contamination, but it can determine tolerable CSF blood contamination, especially for biomarker studies in the field of neurodegenerative diseases. Furthermore, the system is not generally applicable to all downstream CSF analyses. Especially for the analysis of subarachnoid hemorrhage, the source of blood is important to know: is it an intrathecal hemorrhage or peripheral blood contamination due to traumatic lumbar puncture? The detection of erythrophages or siderophages does not allow a definitive differentiation in this context [3,36]. In line with our results, Schwenkenbecher et al. recently showed that a low amount of blood contamination influences the CSF total protein and CSF/serum albumin quotient, leading to false pathological results. The proportion of false results continuously increased with increasing amounts of blood. For the analyses of intrathecal IgG synthesis, they reported 5000 RBC/μL CSF as being still tolerable, which is, however, 100-fold higher than the recommended cut-off value of 50 RBC/µL (equal to the Combur10-Test^®^ grade 3+) by del Campo et al. for AD- and PD-CSF-based biomarker studies [3,11]. The information from our blood contamination experiments is highly relevant to determining whether blood-contaminated CSF samples are acceptable to be included in proteomics-based biomarker studies. In particular, the application of the Combur10-Test^®^ as an approach for the selection of CSF samples has been confirmed. The Combur10-Test^®^ offers several advantages: (i) cost-effectiveness, (ii) speed, (iii) requirement of little sample volume (50 µL), and (iv) easy to handle.

## 5. Conclusions

The information from our blood contamination experiments is highly relevant to determining whether blood-contaminated CSF samples are acceptable for inclusion in proteomics-based biomarker studies. In particular, the application of the Combur10-Test^®^ as an approach for the selection of CSF samples was confirmed. The Combur10-Test^®^ offers several advantages: (i) cost-effective, (ii) fast, (iii) requires little sample volume (50 µL), and (iv) easy to handle.

## Figures and Tables

**Table 1:** Results from the Combur10-Test Strip analysis for the four investigated CSF samples (CSF 1–4).

Sample	1%	0.1%	0.01%	0.001%	0%
CSF 1	++++	++++	+++	++	neg
CSF 2	++++	++++	++++	+++	++
CSF 3	++++	++++	+++	++	neg
CSF 4	++++	++++	+++	++	neg

Each sample was present with five different blood levels including 0% (reference sample), 0.001%, 0.01%, 0.1%, and 1%. All samples were visually evaluated according to the manufacturer’s instructions and the corresponding blood grading of each sample is listed including negative (neg, no blood), 1+ (+), 2+ (++), 3+ (+++), and 4+ (++++).

**Table 2:** Categorization of blood contamination in CSF. For each method, five specific contamination levels were selected on the basis of visual read out (for the Combur10-Test^®^ strips), detection of specific blood proteins (for LC-MS analysis), and defined hemoglobin concentrations (for ELISA).

Contamination value	HB ELISA (ng/mL)	Combur 10-Test®	LC-MS
1	0	negative	no HB or < 5 peptides
10	> 0 to 1,000	1+	HB (≥ 5 peptides)
20	> 1,000 to 15,000	2+ (++)	HB and CAH1
30	> 15,000 to 100,000	3+ (+++)	HB, CAH1 and CATA (≤ 4 peptides)
40	> 100,000	4+ (++++)	HB, CAH1 and CATA (≥ 5 peptides)

**Table 3:** Application of all three methods on independent dataset. Fifteen different CSF samples were analyzed with hemoglobin ELISA, Combur10-Test Strip, and LC-MS. The respective results were classified to the defined contamination levels as listed in Table 2. Samples that were assigned by all three approaches to the same blood level are highlighted in light gray, and samples that were assigned to the same level by two approaches are marked in dark gray.

Sample	ELISA	strip	LC-MS
CSF 5	1	1	1
CSF 6	1	1	1
CSF 7	10	1	10
CSF 8	1	1	1
CSF 9	10	1	1
CSF 10	10	10	10
CSF 11	20	20	20
CSF 12	20	20	20
CSF 13	20	10	10
CSF 14	10	10	10
CSF 15	20	40	40
CSF 16	20	20	20
CSF 17	20	20	20
CSF 18	30	40	40
CSF 19	20	40	30

## Data Availability

The mass spectrometry proteomics data have been deposited to the ProteomeXchange Consortium via the PRIDE (19) partner repository with the dataset identifier PXD014515 and 10.6019/PXD014515.

## Supplementary Materials

The following are available online at https://www.mdpi.com/2073-4409/9/2/370/s1, Figure S1: Quantitative impact of blood contamination on the global proteome of CSF 2 (A), CSF 3 (B) and CSF 4 (C). Table S1: Hemoglobin concentration and contamination level of the 15 clinical CSF samples (CSF 5–19). Table S2: Results from the Combur10-Test Strip analysis and the corresponding contamination level of the 15 clinical CSF samples (CSF 5–19). Table S3: LC-MS based identification of the selected blood proteins in the 15 clinical CSF samples (CSF 5–19) and the corresponding contamination level.

## Submission Details

Project Name: Blood contamination in CSF and its impact on quantitative analysis of alpha-synuclein. Project accession: PXD014515. Project DOI: 10.6019/PXD014515. Reviewer account details: Username: reviewer97801@ebi.ac.uk; Password: zutCzkAI
